# Associations between Stigma, Cognitive Appraisals, Coping Strategies and Stress Responses among Japanese Women Undergoing Infertility Treatment

**DOI:** 10.3390/healthcare10101907

**Published:** 2022-09-29

**Authors:** Rie Yokota, Tsuyoshi Okuhara, Hiroko Okada, Eiko Goto, Keiko Sakakibara, Takahiro Kiuchi

**Affiliations:** 1 Department of Health Communication, Graduate School of Medicine, The University of Tokyo, Tokyo 113-8655, Japan; 2 Department of Health Communication, School of Public Health, The University of Tokyo, Tokyo 113-8655, Japan; 3 Department of Social Psychology, Faculty of Sociology, Toyo University, Tokyo 112-8606, Japan

**Keywords:** infertility, stigma, woman, cognitive appraisal, coping strategy, stress response, mental health, health communication

## Abstract

The number of infertility treatment cycles in Japan is the highest worldwide. Studies have shown that stigma is a predictor of stress-related symptoms including anxiety and depression in women undergoing infertility treatment. Stress management to prevent stress-related symptoms may be crucial; however, few studies have examined the model of stigma and stress responses. Based on the stress-coping model, we hypothesized that stigma threatens the identity of such women and that coping failure increases stress responses. We aimed to explore the role of cognitive appraisals and coping strategies as mediators of the association between the stigma of infertility and stress responses. In December 2021, we conducted a cross-sectional study in Japan, in which 254 women undergoing infertility treatment completed a web-based survey. Hierarchical multiple regression analysis was conducted to analyze the associations between stigma, cognitive appraisals, coping strategies, and stress responses. The results showed that explanatory power increased with each additional variable in the following order: stigma, cognitive appraisals, and coping. Participants with a high level of stigma evaluated it as threatening, and used self-blame and venting coping strategies, and showed higher stress responses. Conversely, participants who used positive reframing coping strategies exhibited lower stress responses. Based on this, effective strategies to address stigma and stress responses are necessitated.

## 1. Introduction

Infertility refers to an ailment of the reproductive system that does not result in a clinical pregnancy despite 12 months of regular unprotected sexual intercourse [1,2]. It is estimated that 48 million couples and 186 million individuals are infertile worldwide [3]. According to a survey conducted in 2015 in Japan, 18.2% of couples and 28.2% of childless couples have, either currently or in the past, undergone medical treatment for infertility or infertility testing [4]. 56,617 births in Japan were made through advanced assisted reproductive technologies (i.e., in vitro fertilization (IVF) and intracytoplasmic sperm injection (ICSI)) in 2017 [5]. In the same year, the number of infertility treatment cycles (448,210 cycles) in Japan was the highest worldwide in terms of utilization [6,7].

Women undergoing assisted reproduction treatment experience more psychological distress, including anxiety and depression, than those who conceive naturally [8]. Infertile women exhibit psychological symptoms comparable to those of patients with cancer, cardiac rehabilitation and hypertension [9,10,11]. In Japan, women undergoing infertility treatment are reported to suffer from high levels of anxiety and depression [6,12,13]. In our earlier study, 51.1% of women undergoing infertility treatment were classified as anxious or suspected and 54.0% as depressed or suspected [13]. Psychological stress because of infertility can have many possible causes [12,14,15,16,17,18,19]. Moreover, the cause of infertility may be unidentifiable, the duration of treatment may be unknown, and financial stress may also be involved [12,14,15,18,19].

Furthermore, the stigma of infertility is negatively associated with mental health, leading to anxiety, depression, social avoidance, and low quality of life [13,20,21,22,23,24,25,26,27,28,29,30,31,32,33,34,35,36]. Stigma is a socially constructed process wherein a group with certain characteristics is labeled with socially unfavorable attributes and, in turn, is slighted by the general public by virtue of attributes or behaviors socially deemed undesirable exhibited by that group [37]. Negatively labeled by people, other than the stigmatized group, as deviant to social norms and expectations, stigmatized individuals internalize these labels [38,39]. 

In Japan, the traditional value of “having children after marriage” remains deeply entrenched, and infertile women having internalized such values, become distressed [40,41]. A survey conducted in 2015 showed that the percentages of respondents who answered that married couples should have children were 67.4% for unmarried women and 66.6% for married women [4]. In this study, we examined the level of stigma among women undergoing infertility treatment in Japan, which was higher than that in Turkey and China, with the limitation that the inclusion criteria were different [42]. Furthermore, the findings of our previous study demonstrated that among the women undergoing infertility treatment in Japan, those who experienced strong stigma showed symptoms of anxiety and depression [13].

Stress-related symptoms including anxiety and depression are caused by continued exposure to a stressor [43,44,45]. Therefore, it is critical to examine the stress process and management [46]. Regarding the process of stress, Lazarus and Folkman have proposed a “Transactional Model of Stress and Coping” focusing on the cognitive appraisal of the stressor and coping [46,47]. First, the stimulus is evaluated as a commitment, effect, or threat to oneself [46,47,48]. Next, a coping strategy is selected, and consequently, stress responses occur. Coping is a cognitive and behavioral effort made in response to internal and external demands from the environment [46,47]. Based on these stress-coping models, several theories assume that stigma is a stressor [49,50,51,52,53,54]. The Major’s model of stigma-induced identity threat is one in which perceived stigma and sensitivity to stigma lead to cognitive appraisals of identity as being threatened, failure to cope, and stress responses [49]. Thus, stress management to prevent stress-related symptoms while considering the stigma of infertility may be important; however, to our knowledge, studies have not examined stigma and stress response processes in patients undergoing infertility treatment.

Few studies have examined cognitive appraisals of women undergoing infertility treatment. According to a U.S. study, there were significantly positive indirect effects of social pressure for motherhood on distress through cognitive appraisal [23]. Studies on mental illness have shown that stigma is a predictor of cognitive appraisal (stigma stress) and, in turn, low well-being [53]. However, cognitive appraisals mediating the relationship between stigma and stress responses after recalling the stigma stressors among infertile women have not yet been tested. Regarding coping among such women, a Turkish study indicated that problem-focused and emotion-focused coping were negatively correlated with hopelessness [55]. Coping has also been found to mediate the association between stigma and quality of life regarding infertility (FertiQOL) in a Chinese study [26]. The indirect effects of stigma on FertiQOL were significantly negative through active avoidance (i.e., self-distraction, behavioral disengagement, and self-blame), active confronting (i.e., using emotional support, instrumental support, active coping, and venting), and passive avoidance (i.e., religion and acceptance) [26]. There were significantly positive indirect effects of stigma on FertiQOL through meaning-based coping (i.e., positive reframing) [26]. However, it is unclear which coping mechanism is mediated by the association between stigma and stress responses. In our previous study, we examined the association between stigma and anxiety/depression [13]; however, examining the process of stigma-induced stress would suggest a direction toward effective interventions. That is, if stigma, cognitive appraisal, and coping were examined for their paths to stress responses, it would provide suggestions for psychological interventions during infertility treatments.

**Figure 1:**
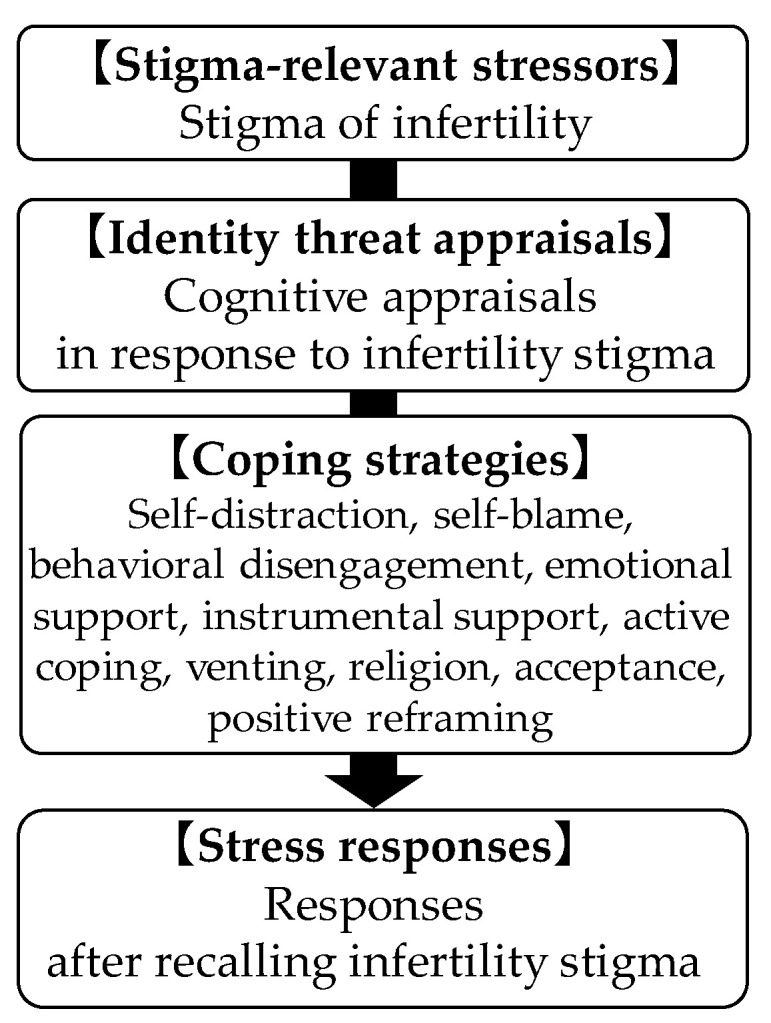
Conceptual hierarchical framework of predictors of stress responses.

This study aimed to examine the role of cognitive appraisals and coping strategies as mediators of the association between the stigma of infertility and stress responses among women undergoing infertility treatment. Referring to Lazarus and Folkman’s and Major’s models as well as previous studies, we tested the following hypotheses among women undergoing infertility treatment in Japan (Figure 1). Mediation analyses were performed to identify the pathways of the relationship between stigma and stress responses.

**H1:**  Women undergoing infertility treatment with a high level of stigma evaluate it as stressful (commitment, appraisal of effect, and appraisal of threat) and use coping strategies such as self-distraction, self-blame, behavioral disengagement, emotional support, instrumental support, active coping, venting, religion, and acceptance, show higher stress responses. Conversely, women who use positive reframing strategies exhibit lower stress responses.

**H2:**  All predictor variables significantly explain the variance in stress responses, with the inclusion of stigma, cognitive appraisal, and coping strategies at each step significantly increasing the results.

## 2. Materials and Methods

### 2.1. Study Design and Setting

This cross-sectional study was part of our previous study, which developed the Japanese version of the Infertility Stigma Scale [42]. Convenience sampling was used to recruit women undergoing infertility treatment in Japan. An Internet survey was conducted in December 2021, and the responses to the survey were completed online. The participants in the present study were the same as those in the scale development study [42]. As noted in the scale development paper, there were no deleted items in the Infertility Stigma Scale, and no item changes were required [42]. Therefore, the items used in the present study were the same as those used in our previous study [42].

### 2.2. Participants and Procedure

Participants were invited from among those listed in the Japanese survey company’s database. Women between the age of 20 and 59 years residing in Japan were identified from the survey company’s database and offered to complete a survey. Emails regarding the survey were sent to these women and instructions were given to access the website of the survey company if they wanted to participate. Following the login, these women chose this study’s survey and advanced to the screening phase. In total, 100,208 monitors received emails. Among them, 10,000 responded to the screening questions. Those women were selected as participants who met the following criteria in the screening questions: (1) aged 20–59 years, (2) currently receiving infertility treatment (except for women being tested for infertility), (3) experiencing primary infertility, (4) native speakers of the Japanese language, and (5) married (inclusive of de facto marriages). The exclusion criteria were as follows: (1) women with healthcare background or experience and (2) women currently or previously diagnosed with a mental illness. A total of 9734 women were screened during the screening phase.

In total, 266 participants, who consented to participate, completed the online survey. We stopped inviting participants to the survey after 266 participants had completed the survey. We then received the survey data from the survey company. The final part of the survey included the question, “Please choose ‘uncertain’ from the following options”, to ensure that participants had adequately responded to the question. Of the 266 respondents, nine who chose options except “uncertain” were considered lax respondents and were excluded. Three participants (two not undergoing infertility treatment when the survey was conducted, and one pregnant) were excluded because they did not meet the eligibility criteria. Finally, data from 254 participants were analyzed. The sample size for multiple regression analysis must be N > 50 + 8 m (where m is the number of independent variables) [56]. Therefore, a sample size of approximately 242 women was desirable, and with 254 participants, this study had an adequate sample size.

### 2.3. Ethical Considerations

This study was conducted per the Declaration of Helsinki. All study participants provided written informed consent and the study design was approved by the Ethics Committee of the Graduate School of Medicine, the University of Tokyo (Approval number: 2021128NI). 

### 2.4. Measures

In the present study, the independent variable was the stigma of infertility (one variable). The mediating variables were cognitive appraisals (three variables) and coping strategies (10 variables). The dependent variable was stress responses (one variable). The control variables were sociodemographic factors (six variables) and clinical characteristics (four variables). In this study, the stigma of infertility was defined as perceived and self-stigmatized. Cognitive appraisal was defined as the way in which stigma was evaluated. A coping strategy was defined as coping as a dynamic process that changes with the situation. Stress responses were defined as responses to the stigma.

#### 2.4.1. Sociodemographic Factors and Clinical Characteristics

Participants provided the following sociodemographic information: age, education, annual household income, employment status, duration of marriage, and family members living together. The participants also responded to the following clinical characteristics: duration of infertility and infertility treatment, determinism of etiology, and type of infertility treatment.

#### 2.4.2. The Japanese Version of the Infertility Stigma Scale (ISS)

Fu et al. developed the Infertility Stigma Scale (ISS) [57]. This scale evaluates an individual’s perceived and self-stigma [57]. The ISS has been widely used in studies in China and Turkey [22,26,27,28,31,33,34,55]. The scale contains 27 items that can be divided into four subscales: self-devaluation, social withdrawal, public stigma, and family stigma. Responses were recorded on a 5-point scale (1 = totally disagree to 5 = totally agree), and combined scores ranging from 27 to 135 were calculated. Higher scores indicated higher levels of stigma. The Cronbach’s alpha coefficient of the original scale developed by Fu et al. was 0.94 [57]. The Japanese version of the ISS was developed by us on the basis of the scale developed by Fu et al. [42]. We also examined its reliability and validity [42]. Similar to the original scale, the Japanese version of the scale included 27 items and had Cronbach’s alpha coefficient of 0.95 [42].

#### 2.4.3. The Cognitive Appraisal Rating Scale (CARS)

The Cognitive Appraisal Rating Scale (CARS), developed in Japan by Suzuki and Sakano in 1998 [48], was employed to assess cognition related to the stressors of stigma of infertility. The CARS is sufficiently valid and reliable and has been standardized for the Japanese population. The instructions for this scale enabled the stressful situation to be structured as intended. In this study, we examined how women undergoing infertility treatment evaluate themselves in situations of perceived and self-stigma. The following situations were presented to the participants: “How do you perceive situations in which you feel negatively about being different from others in society because of your infertility (situation as responded to in items of the ISS)?” The scale consists of eight items and the responses are recorded on a 4-point scale (0 = not at all to 3 = absolutely right). The CARS can be divided into four subscales (two items each): commitment, appraisal of effect, appraisal of threat, and controllability. Commitment refers to how actively individuals engage in resolving the stigma of infertility. Appraisal of the effect is the extent to which stigma of infertility affects their lives. Appraisal of threat is the extent to which they are threatened by the stigma of infertility. Controllability is the extent to which they feel that they can control the stigma of infertility. Per Suzuki and Sakano, commitment, appraisal of effect, appraisal of threat, and controllability are consistent with Lazarus and Folkman’s dimensions of the challenge, harmful effect, threat, and controllability [47,58]. In this study, primary appraisals (mainly, the degree to which they are perceived as self-relevant or threatening) were the target of the research; therefore, controllability, which is a secondary appraisal (how they can cope with those demands), was excluded from the analyses. Therefore, this study used the subscales of commitment, appraisal of effect, and appraisal of threat. Cronbach’s alpha coefficients for stressors in interpersonal situations in the general adult population were 0.81 for appraisal of effect, 0.64 for appraisal of threat and 0.75 for commitment [48]. However, in this study, Cronbach’s alpha coefficients were 0.90, 0.88, and 0.85 for appraisal of effect, appraisal of threat, and commitment, respectively.

#### 2.4.4. The Japanese Version of the Brief Cope Inventory

The Brief COPE Inventory (Brief COPE) was developed by Carver in 1997 to assess coping strategies [59]. The Brief Cope is a shorter version of the COPE Inventory (COPE), which consists of 60 items. COPE is widely used in studies written in English [60] and in research on infertility [55,61,62,63]. The Brief COPE consists of 28 items and the responses are recorded on a 4-point scale (1 = I have not been doing this at all to 4 = I have been doing this a lot). This scale can be classified into 14 subscales (two items each): self-distraction, active coping, denial, substance use, emotional support, instrumental support, behavioral disengagement, venting, positive reframing, planning, humor, acceptance, religion, and self-blame. The Japanese version was translated by Otsuka in 2008 [64]. Otsuka noted a caveat in using the Japanese version, pointing out that the Brief COPE subscales were established using theoretical construction methods; thus, it is not appropriate to arbitrarily reclassify them or calculate a total score [64]. However, he also mentioned that Brief COPE can only use the subscales necessary for the study’s objectives. Costa et al. recommended that coping should be measured in relation to a specific stressor, using an instrument specifically developed to measure coping with that stressor [65]. Schmidt’s version of the Copenhagen Multicenter Psychosocial Infertility (COMPI) Coping Strategy Scale is a measure of coping used by infertile couples to cope with the pressure of infertility [66,67]. However, it has not been translated into Japanese, validated for internal consistency, and is not widely used in Japan. Therefore, we selected and used the Brief COPE subscales considered relevant to each of the COMPI Coping Strategy Scale items: self-distraction, active coping, emotional support, instrumental support, behavioral disengagement, venting, positive reframing, acceptance, religion, and self-blame. The Cronbach alpha coefficients in Otsuka’s study were 0.46 for self-distraction, 0.47 for active coping, 0.72 for using emotional support, 0.80 for using instrumental support, 0.73 for behavioral disengagement, 0.63 for venting, 0.70 for positive reframing, 0.63 for acceptance, 0.64 for religion and 0.74 for self-blame [64]. The Cronbach alpha coefficients in this study, however, were 0.39 for self-distraction, 0.59 for active coping, 0.72 for using emotional support, 0.83 for using instrumental support, 0.70 for behavioral disengagement, 0.63 for venting, 0.59 for positive reframing, 0.64 for acceptance, 0.51 for religion and 0.78 for self-blame.

#### 2.4.5. The Stress Response Scale-18 (SRS-18)

The Stress Response Scale-18 (SRS-18), developed in Japan by Suzuki et al. in 1997 [68], was employed to assess stress responses after answering questions that recall perceived and self-stigma. This scale is capable of measuring temporary psychological changes induced by stressful situations. The SRS-18 is sufficiently valid and reliable and has been standardized for the Japanese population [68,69]. The scale consists of 18 items and the responses are recorded on a 4-point scale (0 = not at all to 3 = exactly so). The SRS-18 can be separated into three subscales (six items each): depression/anxiety, irritability/anger, and hopelessness. The total scores on this scale range from 0 to 54 and measure the overall psychological stress responses. The SRS-18 has standard scores calculated from a sample of 3841 persons, including the following grades for female adults: low (less than 10 points), mediate (11–1 points), rather high (22–32 points), and high (33 points and above) [68]. The Cronbach alpha coefficients were 0.82 to 0.88 in the general population [68]. However, in this study, the Cronbach alpha coefficient for the total score in this study was 0.95.

### 2.5. Statistical Analyses

Descriptive statistics were calculated for sociodemographic factors and clinical characteristics variables [13]. Two-sample *t*-tests and analysis of variance (one-way ANOVA) were used to examine differences in stress responses according to sociodemographic and clinical variables. After ensuring that the distribution of residuals derived from the assumed model by using the Q-Q plot was approximately normally distributed, hierarchical linear regression was used to examine the associations between stigma, cognitive appraisals, coping strategies, and stress responses. Sociodemographic and clinical variables were previously determined per previous studies using regression analysis [21,22,26,27,55]. A forced entry method was used, that is, the factors to be included in the data were predetermined. Stress responses were measured as dependent variables. In step 1, sociodemographic and clinical variables and measures of stigma were included as independent variables. In addition to these variables, measures of cognitive appraisal were used in step 2. Among these variables, measures of coping strategies were included in step 3. Data were analyzed using R version 4.1.1, and the results were assessed at the significance level of *p* < 0.05.

## 3. Results

### 3.1. Descriptive Statistics

Table 1 shows the participants’ sociodemographic factors, clinical characteristics [13], and their association with stress responses. The mean total SRS-18 score was 20.3 (SD = 13.2). Of the participants, 27.6%, 28.7%, 24.0%, and 19.7% were categorized into low, mediate, rather high, and high grades, respectively. One-way ANOVA and two-sample t-tests showed significant differences in stress responses (total SRS-18 score) regarding education (*p* = 0.014) and living with parents (*p* = 0.005). No significant differences in the SRS-18 scores were found for other sociodemographic factors and clinical characteristics.

### 3.2. Hierarchical Linear Regression Analysis

Table 2 shows the regression results for the associations of stigma, cognitive appraisals, and coping strategies with stress responses, after controlling for sociodemographic factors and clinical characteristics. In step 1, stigma was added as an independent variable after controlling for sociodemographic factors and clinical characteristics. The hierarchical linear regression results showed that sociodemographic factors, clinical characteristics, and stigma, tested in step 1, explained 47.5% of the variance in stress responses. A significant association was found between higher stigma levels and stress responses (standardized β = 0.031; *p* < 0.001). In step 2, we included cognitive appraisals related to stressors of the stigma of infertility (commitment, appraisal of effect, and appraisal of threat) as independent variables. The results showed that cognitive appraisals related to stressors of the stigma of infertility, tested in step 2, explained an additional 5.5% (*p* < 0.001) of the variance in stress responses. There was a significant association between appraisal of threat and stress responses (standardized β = 0.134; *p* < 0.001). In step 3, coping strategies (self-distraction, active coping, emotional support, instrumental support, behavioral disengagement, venting, positive reframing, acceptance, religion, and self-blame) were included as independent variables. The results of the hierarchical linear regression showed that coping strategies, tested in step 3, explained an additional 7.8% (*p* < 0.001) of the variance in stress responses. Finally, significant relationships of stress responses were found with venting (standardized β = 0.106; *p* = 0.004), positive reframing (standardized β = −0.079, *p* = 0.026), and self-blame (standardized β = 0.144; *p* < 0.001). The final model explained 60.8% of variance in the stress responses of the participants. None of the variables had a variance inflation factor greater than 10 at any step, and no multicollinearity was observed.

## 4. Discussion

We examined the role of cognitive appraisals and coping strategies as mediators of the association between stigma and stress responses among women undergoing infertility treatment, using hierarchical multiple regression analysis. H1 was partly supported by the results of this study. Women undergoing infertility treatment with a high level of stigma, evaluated it as a threat, and used coping strategies such as self-blame and venting, had higher stress responses. Conversely, women who used the positive reframing coping strategy showed lower stress responses. Other cognitive appraisals and coping strategies were not found to be associated with stress responses, which is inconsistent with our hypothesis. H2 was supported by the results of this study. Overall, the explained variance in the results significantly increased for stigma, cognitive appraisals, and coping strategies. 

In this study, the mean stress response rate after recalling situations of self-stigma and perceived stigma was 20.3 (SD = 13.2). The mean stress response of women in general was 15.81 (SD 11.12), according to Suzuki’s study [68]. The period in which this study was conducted coincided with the coronavirus disease (COVID-19) pandemic, and this may have increased the level of stress among Japanese people [71]. However, the stress response level among women undergoing infertility treatment after recalling the stigma of infertility was considerably higher than that of women in general. Therefore, effective interventions aimed at stress management among infertile women are required.

The hierarchical linear regression results in step 1 showed that perceived stigma and self-stigma had a positive association with stress responses. That is, women undergoing infertility treatment with a higher level of stigma showed higher stress responses after recalling the stigma of infertility. These results are consistent with those of a study that examined a stress-coping model of mental illness stigma [52]. The meaning-making of the infertility experience may largely include a stigmatizing process that can be painful [40]. In Japan, some infertile women consider becoming a mother post marriage as a natural path for women. Those who are childless may be stigmatized as deviants and feel an inability to escape this stigmatized condition [40,41]. Therefore, interventions are necessitated to reduce the stigma experienced by such women.

Concerning cognitive appraisals, the hierarchical linear regression results in step 2 showed that appraisal of threat to stigma-related stressor had a positive association with stress responses. That is, participants who evaluated stigma as a threat showed higher stress responses after recalling the stigma of infertility. This result is consistent with Major’s model of stigma-induced identity threat [49]. Threats arise when the demands of a self-relevant situation are assessed as being beyond one’s resources to meet those demands [49]. To prevent infertile women from appraising threats in situations where they feel stigmatized, efforts must be made to address perceptions of stigma among infertile women and improve situations in which infertile women are devalued. Reducing appraised threats requires psychoeducation and cognitive therapy. For example, narrative enhancement and cognitive therapy would harness the effects of self-narratives on one’s sense of self and identity [72,73,74], experimental learning, positive change in self-experience, cognitive skill acquisition, enhancement of hope, and coping/emotional change. Moreover, interventions to mitigate the legitimacy of discrimination may also be critical [75]. To improve situations in which infertile women are devaluated, anti-stigma campaigns should also be fostered to reduce the endorsement of such stigmatizing stereotypes against infertile women [76]. For instance, counterframing strategies to dispel stigmatizing frames and present a redefinition of infertility may be effective [77]. A frame refers to a perspective that emphasizes particular aspects while ignoring the rest of the problem [77]. There is a need for conveying such messages through media that weaken the stigmatizing narrative or offer the public a new way of looking at infertile women [76,77]. Thus, to prevent infertile women from appraising their identities as threatened, interventions are required to recognize both infertile women who are stigmatized as well as the general public who stigmatize them.

Regarding coping strategies, the hierarchical linear regression results in step 3 showed that participants who used venting as a coping strategy showed higher stress responses. The utilization of emotional and instrumental support was not significantly associated with stress responses. Concerning venting, active confronting coping strategies coincided with the results of a previous study that showed a negative moderating role between stigma and FertiQOL [26]. Infertile women are more likely to tell about their infertility than men; however, they may experience negativity by disclosing it through emotional expression [24]. Infertile women perceive the attitudes of those around them to be negative and unsupportive [78,79]. In situations where they feel stigmatized about infertility, they may express their feelings to those around them and experience negativity, which in turn may heighten their stress responses. Regarding the use of emotional and instrumental support, social support did not result in a significant difference in stress responses. This may be explained by the difference between the received and perceived support. The coping scale used in this study was also supported. In general, received support is either not associated with health or negatively correlated with it [80]. This could be because of a variety of factors, including mismatches between stressors and support types and the quality of relationships [80]. For perceived social support, one study showed that stigma and depression were negatively correlated with perceived social support [27]. A different result might have been obtained if we had examined the association with perceived social support. Infertile women may need a place where they feel safe to confide in their infertility and express their feelings.

The results showed that participants who used self-blame coping strategies exhibited higher stress responses. Active avoidance coping strategies coincided with the results of a previous study that showed a negative moderating role between stigma and FertiQOL [26]. It has been suggested that infertile women are negatively labeled by people as deviant from social norms, and they may internalize these values, blaming themselves for their inability to have children, thus increasing their stress responses. 

According to our findings, participants who used the coping strategy of positive reframing showed lower stress responses. Meaning-based coping strategies coincided with the results of a previous study that showed a positive moderating role between stigma and FertiQOL [26]. In other words, infertile women can cope with infertility by finding other goals and positively reframing their infertility. Based on the results of these coping strategies, narrative reinforcement and cognitive therapy interventions, such as psychoeducation on positive interpretation of one’s experience, are required [72,73,74]. To encourage infertility patients to use coping strategies such as positive reframing, mass media would need to reinforce messages for infertile women to have other goals and give infertility a positive alternative meaning. This is because people pay attention to the cues presented by others in various daily encounters and read the meaning of communication, which is reinforced through the cues presented by the mass media and builds a framework for later behaviors [81,82].

It should be noted the present study contains several limitations. First, this study was performed during the period of the COVID-19 pandemic. Consequently, the stress responses may have been higher than that during the normal unaffected period, as the COVID-19 pandemic had affected mental health [71]. Therefore, similar studies should be conducted once the effects of the COVID-19 pandemic have decreased. Second, because this survey was administered to those listed in the survey company’s database, the interpretation of the data results must consider participant selection and sampling biases. Regarding representativeness, participants in this study may have had different attributes than those of the Japanese population undergoing fertility treatment. Furthermore, participants were women who received emails from the survey company and willingly responded to the survey of this study. Hence, the generalizability of the results of this study to all Japanese women undergoing infertility treatments should be treated with caution. However, the bias of social desirability may have been mitigated, unlike in the recruitment of participants from hospitals and clinics. Fourth, the Brief COPE used in this study was not specific to coping strategies for infertility-related pressures. Moreover, it is not a measure of coping strategies related to the stigma of infertility. Since coping strategies vary according to the stressor [65], we may not have properly assessed coping strategies for the stigma of infertility. Future studies should develop a Japanese version of the COMPI Coping Strategy Scale. Alternatively, a scale should be developed to measure coping strategies for the stigma surrounding infertility. Fifth, the cross-sectional design of this study made it impossible to determine the direction of the tested associations. However, the associations were derived from stigma models [49]. Consequently, future studies with longitudinal data are required to preclude the potential for reverse effects. Despite these limitations, this study is the first to examine the role of cognitive appraisals and coping strategies as moderators of the association between stigma and stress responses in women undergoing infertility treatment.

## 5. Conclusions

In this study, we found that women undergoing infertility treatment with a high level of stigma evaluated it as a threat, and those who used coping strategies such as self-blame and venting showed higher stress responses. Conversely, women who used positive reframing coping strategies exhibited lower stress responses. We also found that the explained variance in the results significantly increased for stigma, cognitive appraisals, and coping strategies. Women undergoing infertility treatment may feel stigmatized, have a threatened identity, fail to cope, and experience heightened stress responses. These results suggest that psychoeducational interventions should be undertaken to harness the effects of self-narratives on one’s sense of self and identity. For instance, psychoeducation that encourages positive reframing and focuses on the positive aspects of the infertility experience, without self-blame, is recommended. Moreover, a safe place where people can express their feelings regarding the stigma of infertility is indispensable. Fostering anti-stigma campaigns to mitigate the popularity of such stigmatizing stereotypes against infertile women is also required.

## Figures and Tables

**Table 1:** Participants’ sociodemographic factors, clinical characteristics, and their association with stress responses. (*n* = 254).

Item		N	%	Stress (SRS-18)		*p* Value
				Mean	SD ^a^	
Age (Mean, SD: 35.9, 5.5)						0.242 ^b^
	20–29	36	14.2	23.4	13.0	
	30–39	147	57.9	20.3	13.0	
	≥40	71	28.0	18.8	13.5	
Education						0.014 *^b^
	Less than high school	1	0.4	0.0	NA	
	High school graduate	43	16.9	23.9	11.9	
	Vocational school graduate	33	13.0	19.5	11.2	
	Junior college or technical college graduate	44	17.3	22.2	14.7	
	University graduate	125	49.2	19.6	13.2	
	Graduate school graduate	8	3.1	8.0	10.1	
Annual household income ^d^						0.208 ^b^
	Less than JPY 2,000,000 (low)	12	4.7	26.1	14.7	
	JPY 2,000,000 to JPY 4,000,000 (low)	38	15.0	23.2	13.0	
	JPY 4,000,000 to JPY 6,000,000 (medium)	72	28.3	20.4	11.9	
	JPY 6,000,000 to JPY 8,000,000 (high)	59	23.2	20.3	14.7	
	JPY 8,000,000 to JPY 10,000,000 (high)	33	13.0	16.7	11.9	
	More than JPY 10,000,000 (high)	40	15.7	18.7	13.4	
Employment status						0.903 ^c^
	Unemployed	80	31.5	20.2	13.7	
	Employed	174	68.5	20.4	13.0	
Duration of marriage (Mean, SD: 4.7, 3.8)						0.863 ^c^
	<5 years	171	67.3	20.2	13.4	
	≥5 years	83	32.7	20.5	12.9	
Living with your parents						0.005 **^c^
	No	239	94.1	19.7	13.0	
	Yes	15	5.9	30.0	11.9	
Duration of infertility (Mean, SD: 3.3, 2.9)						0.602 ^c^
	<5 years	205	80.7	20.1	12.9	
	≥5 years	49	19.3	21.3	14.4	
Duration of infertility treatment (Mean, SD: 2.3, 2.4)						0.993 ^c^
	<3 years	190	74.8	20.3	13.3	
	≥3 years	64	25.2	20.3	13.0	
Determinant of etiology						0.181 ^c^
	No	117	46.1	21.5	13.2	
	Yes	137	53.9	19.3	13.2	
Treatment for infertility						0.363 ^c^
	Other	134	52.8	21.0	13.0	
	IVF and ICSI	120	47.2	19.5	13.4	
Total		254		20.3	13.2	

* *p* < 0.05, ** *p* < 0.01 ^a^ Standard deviation. ^b^ One-way ANOVA. ^c^ Two-sample *t*-test ^d^ The median annual household income in Japan was JPY 4,400,000, and the mean annual household income was JPY 5,643,000 in 2020 [70]. Therefore, we defined medium as between JPY 4,000,000 and JPY 6,000,000, low as less than JPY 4,000,000, and high as more than JPY 6,000,000.

**Table 2:** Hierarchical linear regression predicting stress responses (SRS-18). (*n* = 254).

	Step1					Step2					Step3				
	B	SE ^a^	Stdβ ^b^	t	*p*	B	SE ^a^	Stdβ ^b^	t	*p*	B	SE ^a^	Stdβ ^b^	t	*p*
(Intercept)	20.315	0.613		33.139	<0.001	20.315	0.584		34.812	<0.001	20.315	0.544		37.343	<0.001
Age (years)	−0.206	0.136	−0.016	−1.509	0.133	−0.208	0.130	−0.016	−1.594	0.112	−0.140	0.126	−0.011	−1.111	0.268
Education ^c^	−1.417	0.544	−0.108	−2.606	0.010	−1.028	0.524	−0.078	−1.961	0.051	−1.092	0.495	−0.083	−2.207	0.028
Annual household income ^d^	−0.245	0.484	−0.019	−0.507	0.613	−0.445	0.463	−0.034	−0.961	0.338	−0.194	0.440	−0.015	−0.440	0.660
Employment status ^e^	−0.570	1.378	−0.043	−0.414	0.679	−0.710	1.318	−0.054	−0.539	0.590	−0.476	1.254	−0.036	−0.379	0.705
Duration of marriage (years)	0.063	0.300	0.005	0.210	0.834	0.047	0.289	0.004	0.163	0.870	0.124	0.274	0.009	0.454	0.650
Living with your parents ^f^	8.914	2.626	0.676	3.394	<0.001	7.592	2.522	0.576	3.010	0.003	8.029	2.366	0.609	3.393	<0.001
Duration of infertility (years)	−0.253	0.510	−0.019	−0.496	0.620	−0.244	0.487	−0.019	−0.501	0.617	−0.322	0.455	−0.024	−0.706	0.481
Duration of infertility treatment (years)	−0.561	0.506	−0.043	−1.108	0.269	−0.336	0.485	−0.026	−0.693	0.489	−0.327	0.459	−0.025	−0.712	0.477
Determinant of etiology ^g^	−3.612	1.262	−0.274	−2.863	0.005	−3.183	1.206	−0.242	−2.639	0.009	−3.044	1.139	−0.231	−2.672	0.008
Treatment for infertility ^h^	0.648	1.399	0.049	0.463	0.644	0.359	1.334	0.027	0.269	0.788	0.685	1.271	0.052	0.539	0.590
Stigma (ISS) ^i^	0.405	0.030	0.031	13.271	<0.001	0.287	0.037	0.022	7.748	<0.001	0.227	0.039	0.017	5.862	<0.001
Commitment (CARS) ^j^						0.142	0.543	0.011	0.262	0.794	−0.174	0.529	−0.013	−0.329	0.742
Appraisal of effect (CARS) ^j^						0.361	0.560	0.027	0.645	0.519	0.475	0.532	0.036	0.892	0.373
Appraisal of threat (CARS) ^j^						1.767	0.413	0.134	4.284	<0.001	0.969	0.419	0.074	2.310	0.022
Self-distraction (Brief COPE) ^k^											−0.338	0.505	−0.026	−0.669	0.504
Active coping (Brief COPE) ^k^											−0.257	0.558	−0.020	−0.461	0.645
Using emotional support (Brief COPE) ^k^											0.282	0.515	0.021	0.548	0.584
Using instrumental support (Brief COPE) ^k^											−0.142	0.494	−0.011	−0.287	0.775
Behavioral disengagement (Brief COPE) ^k^											−0.055	0.457	−0.004	−0.120	0.905
Venting (Brief COPE) ^k^											1.399	0.477	0.106	2.935	0.004
Positive reframing (Brief COPE) ^k^											−1.047	0.467	−0.079	−2.245	0.026
Acceptance (Brief COPE) ^k^											−0.158	0.530	−0.012	−0.299	0.766
Religion (Brief COPE) ^k^											0.731	0.398	0.055	1.837	0.068
Self-blame (Brief COPE) ^k^											1.901	0.463	0.144	4.106	<0.001
R^2^	0.475					0.530					0.608				
Adjust R^2^	0.451					0.502					0.567				
F (*p* value)	19.87	(*p* < 0.001)				19.23	(*p* < 0.001)				14.82	(*p* < 0.001)			
ΔR^2^ (*p* value)	0.475					0.055	(*p* < 0.001)				0.078	(*p* < 0.001)			

^a^ Standard error. ^b^ Standardized β. ^c^ 0 = less than high school, 1 = high school graduate, 2 = vocational school graduate, 3 = junior college or technical college graduate, 4 = university graduate, 5 = graduate school graduate. ^d^ 0 = less than JPY 2,000,000, 1= JPY 2,000,000 to JPY 4,000,000, 2= JPY 4,000,000 to JPY 6,000,000, 3 = JPY 6,000,000 to JPY 8,000,000, 4 = JPY 8,000,000 to JPY 10,000, 000. 5 = more than JPY 10,000,000. ^e^ 0 = unemployed, 1 = employed. ^f^ 0 = no, 1 = yes. ^g^ 0 = no, 1 = yes. ^h^ 0 = other than IVF and ICSI, 1 = IVF and ICSI. ^i^ 27–135. ^j^ 0–6. ^k^ 2–8 4.

## Data Availability

The data supporting the findings of this study are available from the corresponding author, R.Y., upon reasonable request. The data were not publicly available because of ethical considerations.
